# Neodymium Recovery by Chitosan/Iron(III) Hydroxide [ChiFer(III)] Sorbent Material: Batch and Column Systems

**DOI:** 10.3390/polym10020204

**Published:** 2018-02-19

**Authors:** Hary Demey, Byron Lapo, Montserrat Ruiz, Agustin Fortuny, Muriel Marchand, Ana M. Sastre

**Affiliations:** 1 Department of Chemical Engineering, Universitat Politècnica de Catalunya, ETSEIB, Diagonal 647, 08028 Barcelona, Spain; Byron.lapo@upc.edu (B.L.); Ana.maria.sastre@upc.edu (A.M.S.); 2 Commissariat à l’Energie Atomique et aux Energies Alternatives, CEA/DRT/LITEN/DTBH/LTB, 17 rue des Martrys, 38054 Grenoble, France; muriel.marchand@cea.fr; 3 School of Chemical Engineering, Universidad Técnica de Machala, UACQS, 070151 Machala, Ecuador; 4 Department of Chemical Engineering, Universitat Politècnica de Catalunya, EPSEVG, Av. Víctor Balaguer, s/n, 08800 Vilanova i la Geltrú, Spain; Montserrat.ruiz@upc.edu (M.R.); agustin.fortuny@upc.edu (A.F.)

**Keywords:** boron, chitosan, iron(III) hydroxide, neodymium, sorption

## Abstract

A low cost composite material was synthesized for neodymium recovery from dilute aqueous solutions. The in-situ production of the composite containing chitosan and iron(III) hydroxide (ChiFer(III)) was improved and the results were compared with raw chitosan particles. The sorbent was characterized using Fourier transform infrared spectroscopy (FTIR) and scanning electron microscopy-energy dispersive X-ray analyses (SEM-EDX). The equilibrium studies were performed using firstly a batch system, and secondly a continuous system. The sorption isotherms were fitted with the Langmuir, Freundlich, and Sips models; experimental data was better described with the Langmuir equation and the maximum sorption capacity was 13.8 mg·g^−1^ at pH 4. The introduction of iron into the biopolymer matrix increases by four times the sorption uptake of the chitosan; the individual sorption capacity of iron (into the composite) was calculated as 30.9 mg Nd/g Fe. The experimental results of the columns were fitted adequately using the Thomas model. As an approach to Nd-Fe-B permanent magnets effluents, a synthetic dilute effluent was simulated at pH 4, in order to evaluate the selectivity of the sorbent material; the overshooting of boron in the column system confirmed the higher selectivity toward neodymium ions. The elution step was carried out using MilliQ-water with the pH set to 3.5 (dilute HCl solution).

## 1. Introduction

Rare earth elements (REEs) are critical due to their importance for many technological applications. The greater reserves of REEs are located in China; being the main world producer (since the 1990s it has been producing roughly 90% of the world’s supply). REEs are massively used in the manufacturing of high-technology devices; the growing demand of the last decade has forced the mining industry of the European Union to search for new mineral sources outside of China (in order to reduce the strong dependence to this country). Additionally, the popular concept of “urban mining” has been generalized in all European countries over the last five years: society is recognizing that resources contained in wastes should be recovered and reused as much as possible [1].

REEs are distributed in moderated concentration in the earth’s crust; the term “rare” originated from the difficult isolation processes on their discovery. Their abundance follows the Oddo–Harking rules: elements with even-atomic number are more stable and more abundant than adjacent odd-atomic elements [2,3]. The most abundant REEs in the earth crust are yttrium (28–70 mg·kg^−1^) and cerium (20–70 mg·kg^−1^) and the less abundant is thulium (0.2–1.0 mg·kg^−1^). Special attention must be paid to neodymium (Nd), which is a key element in the high-tech industries and its applications include the manufacturing of the high strength permanent magnets (Nd-Fe-B), the fabrication of electrical motors for hybrid vehicles, and wind turbine generators [4].

Although the concentration of Nd in the earth’s crust is moderate (12–41.5 mg·kg^−1^), it is also considered as a critical element due to the possible shortage in Chinese export to the EU and USA [5,6]. Therefore, the development of novel technologies for recycling and recovery of neodymium is an important task for government authorities. Currently, there is no a simple method for Nd separation from aqueous effluents. Nevertheless, several methodologies have been used for metal removal from aqueous solutions, such as chemical precipitation [7,8], electrochemical treatment [9,10], membrane technology [11,12], solvent extraction [13], ion-exchange [14,15], and biosorption [16,17,18,19].

Recently, our research group [20] suggested liquid-liquid extraction technique with Cyanex-272 and Cyanex-572 as a good alternative for Nd separation from waste magnets effluents. This process is particularly useful and efficient for the treatment of wastewaters with high metal concentrations; otherwise, it could not be cost-effective for separating traces of metals, due to the involvement of large amounts of organic solvents and a further step for eluent treatment.

Biosorption could be regarded as the most suitable and economical method for recovering metal ions from dilutes effluents [21,22]. Furthermore, the designing of sorbents is crucial for reaching a high performance; the combination of several reactive groups may improve the stability of the resulting material and improve the removal uptake. To achieve this goal, a composite consisting of chitosan and iron(III) hydroxide [ChiFer(III)] was manufactured (in-situ) and evaluated for Nd sorption from aqueous solutions. Previous studies [23] have reported the use of ChiFer(III) material for boron removal from Mediterranean seawater, the sorbent is stable for several sorption–desorption cycles and can be used under conditions with high ion-strength (i.e., seawater); this information is advantageous for the treatment of waste magnet effluents (e.g., Nd-Fe-B).

In comparison with heavy metals, the literature for REEs recovery through the sorption process is still scarce. Several authors have reported the possibility of using biopolymer-based materials; e.g., Wang et al. [24] evaluated the performance of calcium alginate for Nd sorption, and Galhoum et al. [25] used cysteine-functionalized chitosan magnetic nano-based particles for removal of light and heavy rare earth elements from aqueous solutions: cationic species La(III), Nd(III), and Yb(III) can be sorbed by a combination of chelating and anion-exchange mechanisms; and the thermodynamic constants demonstrated the spontaneous and endothermic nature of sorption. Additionally, Zhao et al. [26,27] synthesized two innovative materials based on: i) EDTA-cross-linked β-cyclodextrin (EDTA-β-CD); and ii) polyethylenimine-cross-linked cellulose nanocrystals (PEI-CNC). The sorbents were demonstrated to be selective for Eu(III) and Er(III) recovery from waters, respectively (i.e., the selectivity of EDTA-β-CD follows: Eu(III) over Ce(III) and La(III); and PEI-CNC follows: Er(III) over La(III) and Eu(III)).

Chitosan is a copolymer of glucosamine and *N*-acetyl-d-glucosamine linked by β(1→4) glycosidic bonds [28,29]; its applications are derived from its easy availability (as a renewable resource) and its easy transformation into different configurations: chitosan is considered as the second most abundant biopolymer in the environment after cellulose. This study reports the preparation of chitosan-based composite to improve the handling of Fe(OH)_3_ as sorbent to recover neodymium in a continuous system. The regeneration of the ChiFer(III) material was evaluated with dilute HCl solution (demineralized water at pH 3.5), and the Thomas model [30] was used to fit the experimental data.

## 2. Materials and Methods

### 2.1. Materials

Neodymium solutions were prepared using Nd(NO_3_)_3_·6H_2_O (molecular weight 438.35 g·mol^−1^) provided by Sigma-Aldrich (St. Louis, MO, USA). Iron(III) chloride hexahydrate used for the sorbent preparation was provided by Panreac (Barcelona, Spain). Chitosan was supplied by Aber Technologies (Lannilis, France), and its molecular weight (125,000 g·mol^−1^) was previously reported by Ruiz et al. [31] using size exclusion chromatography (SEC) coupled with light scattering and refractometry. The degree of acetylation determined by Fourier transform infrared (FTIR) spectroscopy was found to be 0.13 (i.e., the deacetylation degree of chitin is 0.87) [32].

### 2.2. Preparation of ChiFer(III) Microspheres

The manufacturing of the sorbent material was slightly improved from that of Demey et al. [23]; the chitosan solution with a concentration of 2.2% *w*/*w*, was prepared by dissolving 30 g of chitosan in 2.2% *w*/*w* acetic acid solution (1350 mL) and stirring for 5 h. Thirty grams of FeCl_3_·6H_2_O powder were mixed in 120 mL of HCl solution (0.5 M) until complete dissolution. The chitosan solution (1350 mL, 2.2% *w*/*w*) was then mixed with the iron(III) solution under vigorous stirring (500 rpm) for 2 h.

The chitosan-iron(III) mixture was added drop-by-drop with a peristaltic pump through a thin nozzle (Ø 2.0 mm) into an aqueous solution of 1 M sodium hydroxide under magnetic stirring to produce microspheres of the ChiFer(III) composite. The resulting beads were kept under stirring for 8 h at room temperature (25 °C) and were then filtered and washed intensively with distilled water to remove the excess of sodium hydroxide from the surface of the sorbent. To compare the effect of the drying method on the kinetic profiles, wet samples of beads were air-dried in a laboratory oven at 45 °C (AD beads), and freeze dried (using a LyoQuest-55, Telstar equipment, São Paulo, Brazil) at 218 K and 0.01 mbar (FD beads). The standard sorbent used in this work was the freeze dried beads with an average diameter of 2.0 mm. The average particle size (Sp) of chitosan was 0.5 mm < Sp < 1 mm (Figure S1 in the supplementary materials section); it was verified through a MASTERSIZER 3000^TM^ equipment from Malvern Instruments Ltd., Worcestershire, UK.

### 2.3. Characterization of Sorbents

#### 2.3.1. Scanning Electron Microscopy

The samples were analyzed using a JEOL JSM 7100F field emission scanning electron microscope (JEOL Ltd., Peabody, MA, USA), a specialized high-performance scanning electron microscope (SEM) with low-vacuum, and high-vacuum modes, capable of analyzing samples under pressures of up to 9.4 × 10^–4^ Pa. The objective lens of the JEOL JSM-7100F does not create a magnetic field around the samples and a high magnification is easily obtained (which improves the study of several types of micro/nano-structures). Thus, magnetic samples can be observed and analyzed without restriction. The microscope is also equipped with an Energy Dispersive X-ray (EDX) spectrometer (INCA 250, Oxford instruments, Oxford, UK) for chemical analysis.

The sorbent samples were analyzed before and after sorption of neodymium from aqueous solutions; EDX-analysis technique was used to detect the main elements present at the surface of the sorbent particles.

#### 2.3.2. FTIR Analyses

Fourier transform infrared (FTIR) analyses were performed using a BRUKER IFS 66 FTIR spectrophotometer (Bruker Optik GmbH, Ettlingen, Germany) equipped with a reflection diamond accessory (platinum ATR), and the spectra were recorded in the range of 4000–400 cm^−1^ with a sample amount of 2 mg of ChiFer(III).

### 2.4. pH Effect

The study of pH-influence on neodymium sorption was performed by mixing 25 mL of dilute metal solution (9.4 mg·L^−1^; i.e., 0.07 mmol·L^−1^) with 25 mg of sorbent (i.e., sorbent dosage, SD 1 g·L^−1^) in 50 mL polyethylene flasks. Proton concentration was adjusted using 0.1 M HCl or 0.1 M NaOH (as required); the evaluated range was set at pH 1–6 (to prevent metal precipitation in all experimental series) and the stirring speed was set at 150 rpm at 20 °C, using an agitator Rotabit, J.P. Selecta (Barcelona, Spain). After 72 h of agitation, the final pH was measured, and 5 mL of solution was filtered and analyzed with 4100 MP-AES instrument (Microwave plasma-atomic emission spectrometer from Agilent technologies, Melbourne, Australia) at the wavelength of 430.35 nm (for neodymium detection).

### 2.5. Equilibrium Sorption

Sorption isotherms were obtained by mixing a known volume of solution (25 mL) at different metal concentrations at selected pH 4 and a fixed mass of sorbent (25 mg; i.e., SD 1 g·L^−1^). After 72 h of contact, the pHs of the solutions were measured and the initial and equilibrium metal concentrations were systematically determined using 4100 MP-AES equipment (Agilent technologies, Melbourne, Australia).

The Langmuir, the Freundlich, and the Sips equations were used to describe the experimental sorption isotherm data [33,34,35,36]:

(1) $$q=\frac{q_{\max}{\mathrm{bC}}_{\mathrm{eq}}}{1+{\mathrm{bC}}_{\mathrm{eq}}}$$

(2) q=KFCeq1n

(3) q=qmaxKSCeq1ns1+KSCeq1ns

where q is the amount of sorbed metal per gram of sorbent at equilibrium (mg·g^−1^), qmax is the maximum adsorption capacity of the sorbent (mg·g^−1^), and Ceq is the equilibrium concentration of the solution (mg·L^−1^). In the Langmuir model (equation 1), b is related to the energy of adsorption (L·mg^−1^), whereas KF and n are the Freundlich adsorption constants, indicative of the relative capacity and the adsorption intensity, respectively; Ks (L·mg^−1^) and ns are the constants of the Sips model [36]. The experiments of equilibrium studies were performed in duplicate to ensure the accuracy of the results (the relative standard deviation obtained was in the order of ±5%).

### 2.6. Influence of Contact Time

The uptake kinetics experiments were performed by adding (under continuous stirring) a known amount of sorbent (SD 0.5 g·L^−1^) to 500 mL of metal solution (i.e., 10–20 mg·L^−1^) at pH 4. Aliquots of solution were withdrawn at different times and filtered over 72 h of contact with the beads. The residual concentration was determined by the 4100 MP-AES instrument. The kinetic profiles were compared by using sorbent beads under the same conditioning (freeze-dried beads; Ø 2.0 mm). The models such as PFORE and PSORE (pseudo-first order and pseudo-second order rate equations) were evaluated to fit the experimental data; the contribution of the resistance to intraparticle diffusion was evaluated by the equation of Weber and Morris, [37]:

Pseudo-first order rate equation (PFORE) [38,39]:

(4) $$\frac{{\mathrm{dq}}_{t}}{dt}=K_{1}\left(q_{1}-q_{t}\right)$$

Pseudo-second order rate equation (PSORE) [40]:

(5) $$\frac{{\mathrm{dq}}_{t}}{{\left(q_{\mathrm{eq}}-q_{t}\right)}^{2}}=K_{2}dt$$

where q_eq_ is the equilibrium sorption capacity (mg·g^−1^), q_t_ is the sorption capacity (mg·g^−1^) at any time t (min), and k_2_ is the pseudo-second order rate constant (g·mg^−1^·min^−1^).The parameters q_eq_ and k_2_ are pseudo-constants depending on the experimental conditions.

Equation of Weber and Morris, [37]:

(6) $$q_{t}=K_{p}t^{1/2}+C$$

where C is the intercept, and K_p_ is the intraparticle diffusion rate constant.

### 2.7. Dynamic Column Testing

The applicability of a dynamic system was tested for evaluating the sorption performance of ChiFer(III) material on neodymium sorption. The glass columns (i.d., Ø 1.8 cm) were filled with hydrated beads (Ø 2.0 mm; 2.4 g in d.w.) until a bed depth of 23 cm (i.e., bed-volume: 58.53 mL). In order to avoid a poor arrangement of the packed-bed, the sorbent was carefully introduced into the columns from the top in a water suspension (typically 1 g·L^−1^). Then, the bottom of the column was kept open (over 2 h) to evacuate the water content.

The metal solution was delivered by up-flow at 20 °C using a peristaltic pump at a flow rate of 0.01 and 0.02 L·h^−1^ (i.e., superficial velocity: 3.77 × 10^−2^ m·h^−1^ and 8.01 × 10^−2^ m·h^−1^ respectively). The tests were performed at pH 4.5 and inlet concentration of 10 mg·L^−1^. After saturation, the packed-bed was eluted upward using dilute HCl solution (demineralized water at pH 3.5) and a flow rate of 0.01 L·h^−1^. Samples of 5 mL were periodically collected for analyzing during sorption and desorption steps using an automatic fraction collector (Gilson FC-203 B, Dunstable, UK).

It is noteworthy that, the breakthrough curve allows the behavior of recovered metal into the sorbent bed to be represented; it is typically plotted as the ratio of effluent concentration to inlet concentration (C_t_/C_0_) as a function of the operational time t, or also as a function of the ratio volume of the influent/volume of the packed-bed (so called bed-volume, BV). The breakthrough and the exhaustion points were set as C_t_/C_0_ = 0.05 and C_t_/C_0_ = 0.95, respectively. The experimental sorption capacity can be obtained from Equation (7):

(7) $$q_{\exp}=\int_{0}^{V_{\mathrm{total}}}\frac{\left(C_{0}-C_{t}\right)}{m}\mathrm{dV}$$

The Thomas model equation [30] was used for describing the theoretical performance of the columns due to its simplicity and adequate accuracy in predicting breakthrough curves. The model can be represented by the following equation for favorable sorption process [41]:

(8) $$\frac{C_{t}}{C_{0}}=\frac{1}{1+\exp\left[K_{T}\left(q_{T}m-C_{0}V\right)/Q\right]}$$

where K_T_ is the Thomas rate constant (L·h^−1^·mg^−1^), m is the mass of sorbent (g), Q is the volumetric flow rate (L·h^−1^), V is the volume of the solution into the column (L), C_t_ is the metal concentration (mg·L^−1^), and q_T_ is the Thomas sorption capacity (mg·g^−1^). The constants of the non-linearized form were obtained by origin 9.0 software (OriginLab Inc., Northampton, MA, USA, 2012).

In addition, a selectivity study was developed by using a binary system (in columns) with an equimolar solution of neodymium and boron (0.1 mmol·L^−1^), in order to approach the real effluent of permanent magnets (Nd-Fe-B). Sorption systems are usually employed as polishing steps at pH 4–6 (as a complement of traditional physical–chemical techniques); therefore metal concentrations are very dilute at these operating conditions, especially iron concentration which is almost negligible and it was not considered in this study.

The separation coefficients of neodymium and boron (R_Nd/B_) were calculated as follows and plotted versus the bed-volume [42]:

(9) $${\mathrm{Kd}}_{i}=\int_{0}^{V_{\mathrm{total}}}\frac{\left(C_{0}-C_{t}\right)}{C_{0}m}\mathrm{dV}$$

(10) $$R_{\mathrm{Nd}/B}=\frac{{\mathrm{Kd}}_{\mathrm{Nd}}}{{\mathrm{Kd}}_{B}}$$

where R_Nd/B_ is the separation coefficient of Nd and B; K_d_ is the distribution coefficient (L·g^−1^); C_o_ is initial metal concentration (mg·L^−1^); C_t_ is the metal concentration at the time t (mg·L^−1^); m is the mass of the sorbent (g); V is the volume of the solution passed into the column (L).

## 3. Results and Discussion

### 3.1. Characterization of Sorbent 

#### 3.1.1. Scanning Electron Microscopy

**Figure 1:**
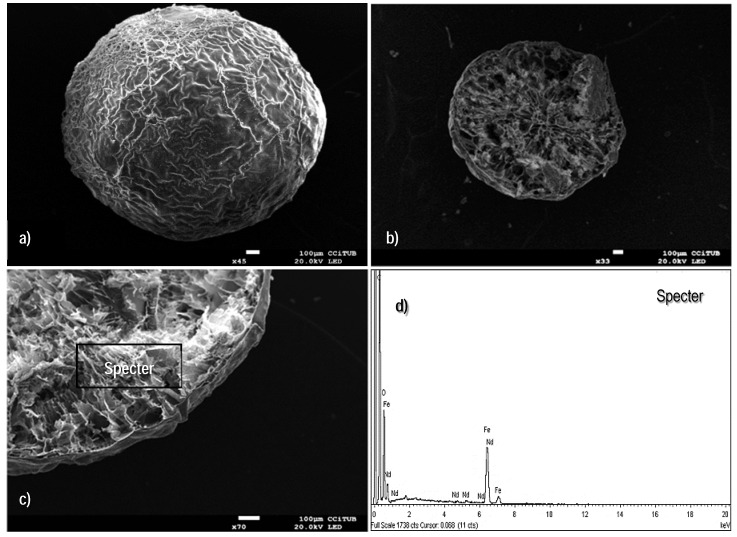
Scanning electron microscopy (SEM) images of the freeze dried (FD) sorbent. (**a**) External surface. (**b**) Cross-section area. (**c**,**d**) Energy dispersive X-ray (EDX) analysis of the cross-section area of the beads.

SEM micrographs were performed for exploring the topography of the sorbent surface, as well as to examine the external and internal structure of the beads configuration. Figure 1a shows the spherical shape of the standard freeze-dried material (average diameter, Ø 2.0 mm); some cavities are found around the entire external surface, which is an indication of the easy accessibility for sorbate molecules into the volume of the beads. The images performed on the cross-section area, corroborate that the internal topography is relatively open (Figure 1b); this is a relevant feature for enhancing the diffusion phenomenon.

EDX-analyses on different zones of the cross-section area (Figure 1c,d) suggest that the main elements of the composite (i.e., oxygen, carbon, and iron) are homogenously distributed in the whole volume of the sorbent. The total amount of iron distributed throughout the dried material was quantified by 4100 MP-AES technique (a known amount of beads was kept under contact with 10 M HNO_3_ solution over 10 h until complete digestion of the organic and mineral components): it was found to be 330 mg of iron per gram of ChiFer(III).

**Figure 2:**
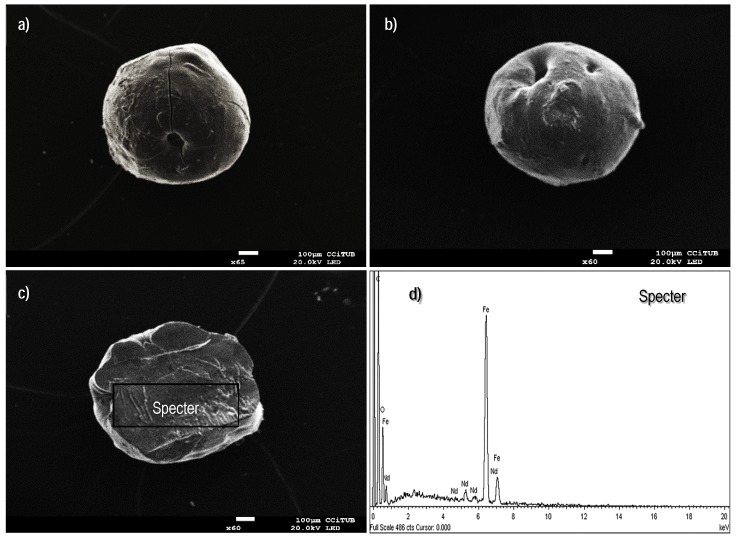
Scanning electron microscopy (SEM) images of the air dried (AD) sorbent. (**a**) External surface. (**b**) Cross-section area. (**c**,**d**) Energy dispersive X-ray (EDX) analysis of the cross-section area of the beads.

The micrographs presented in Figure 1 are in complete agreement with the previous findings reported by Demey et al. [23]; the manufacturing of iron(III) hydroxide through the in-situ coagulation of chitosan solution provides an innovative technique for immobilization of active mineral materials over a polymeric and well distributed network. A second configuration of the sorbent, the so-called xerogel material, was examined for verifying the impact of the drying method on the resulting structure; Figure 2a,b show that the uncontrolled air-drying of the hydrogels leads to shrinkage of the surfaces with depleted performance of mass transfer [43]. The air-dried particles have an average diameter of 0.90 mm; some folds over the entire surface are formed due to water evaporation at ambient pressure conditions, and the cavities or pores (around the folds) appear not to be easy accessible for sorbate ions. Figure 2c shows the cross-section area of the xerogels which differ widely from the freeze-dried beads: it seems to be a closed network; however, the EDX-analysis (Figure 2d) confirmed the content of the main elements, as expected (i.e., oxygen, carbon, and iron).

In this work, the freeze-drying method was preferred over the air-drying technique since the resulting beads could provide a network structure with more easy accessibility, and the regular spherical shape of the particles may improve packaging into the fixed bed (improving the mass transfer of the system). According to Borisova et al. [44] a better storage/conservation of the structures of the hydrogels can be performed by a novel technique based on the supercritical conditions of CO_2_ (scCO_2_), nevertheless the freeze-drying technique is less expensive than scCO_2_ and the resulting dried materials adequately conserve their original structures [45].

**Figure 3:**
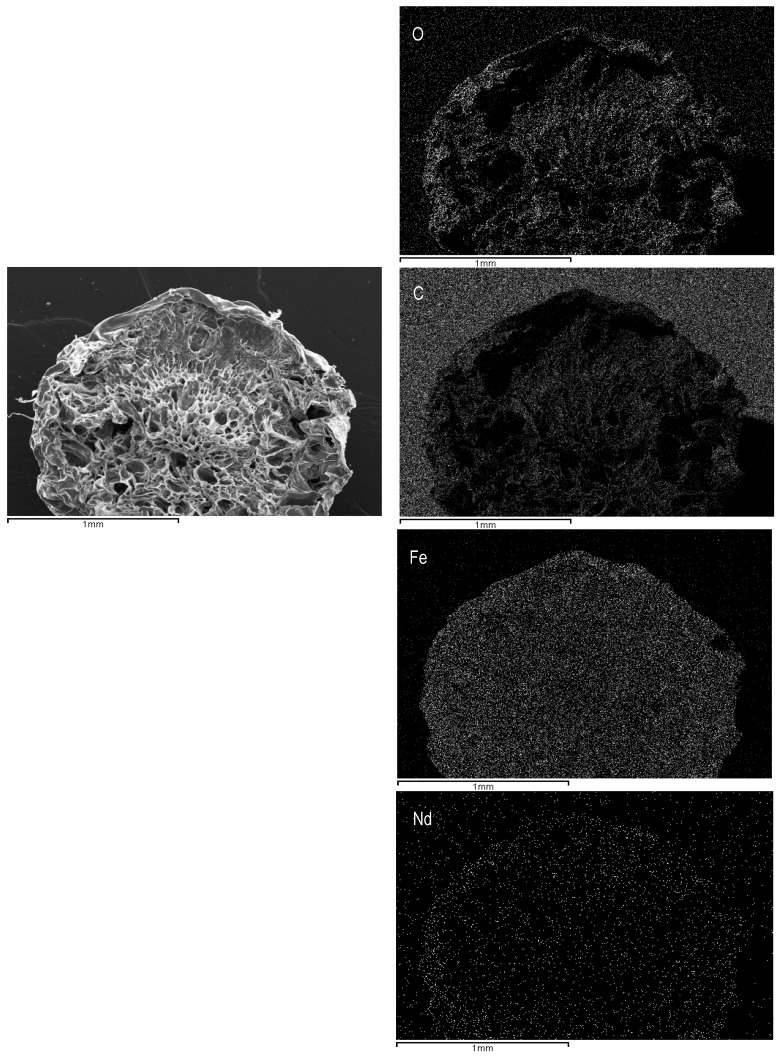
Distribution of the main components on the cross-section area of the ChiFer(III) material after sorption. (Right side: Original structure; left side: Distribution of O: Oxygen; C: Carbon; Fe: Iron; Nd: Neodymium).

Additionally, EDX-analysis were used for examining the cartography on the cross-section area of ChiFer(III) after neodymium removal from aqueous solutions. Figure 3 gives a qualitative representation of the elements contained in the volume of sorbent (such as carbon, oxygen, iron, and neodymium); neodymium appears to be homogenously distributed in the entire volume of the beads, which is useful information for interpreting the kinetic data.

Figure S2 (in the Supplementary Materials Section) shows the characteristics peaks (FTIR analyses) of the ChiFer(III) sorbent; the broad band centered at 3000–3600 cm^−1^ is due mainly to the hydroxyl groups (stretching vibrations of C–OH and Fe–OH) and to the extension vibration of N–H group of the primary amine (–NH_2_) of chitosan [42]. The band at 2885 cm^−1^ is usually assigned to symmetric –CH_2_ groups [28,46]; the bands at 1408 cm^−1^ and 1022 cm^−1^ are attributed to the C–O–C and to the C–O stretching vibrations, respectively [25,47], and the band centered at 1630 cm^−1^ is attributed to the stretching vibration of C=O (carboxylate groups of polysaccharides, [42]). The peak around the band at 898 cm^−1^ was attributed by Wang et al. [28] to the β-d-glucose unit of chitosan. The absorption band at 586 cm^−1^ is due to the Fe–O stretching vibration of Fe_3_O_4_, Shinde et al. [48] found that this band is specially shifted with an increase of Nd concentration (in contact with nickel ferrites material); in this study, the dilute concentrations of neodymium solutions (<0.01 mol·L^−1^) did not lead to verification of this finding.

### 3.2. pH Effect

The pH is one of the most important parameters in the sorption process. The proton concentration influences metal speciation which impacts on metal/sorbent interactions, affecting the sorption performance. The plot of metal distribution of neodymium and iron species at dilute concentrations (1 mmol·L^−1^) is represented in the Supplementary Materials Section (Figure S3); the experiments were conducted to avoid the precipitation phenomenon of neodymium.

**Figure 4:**
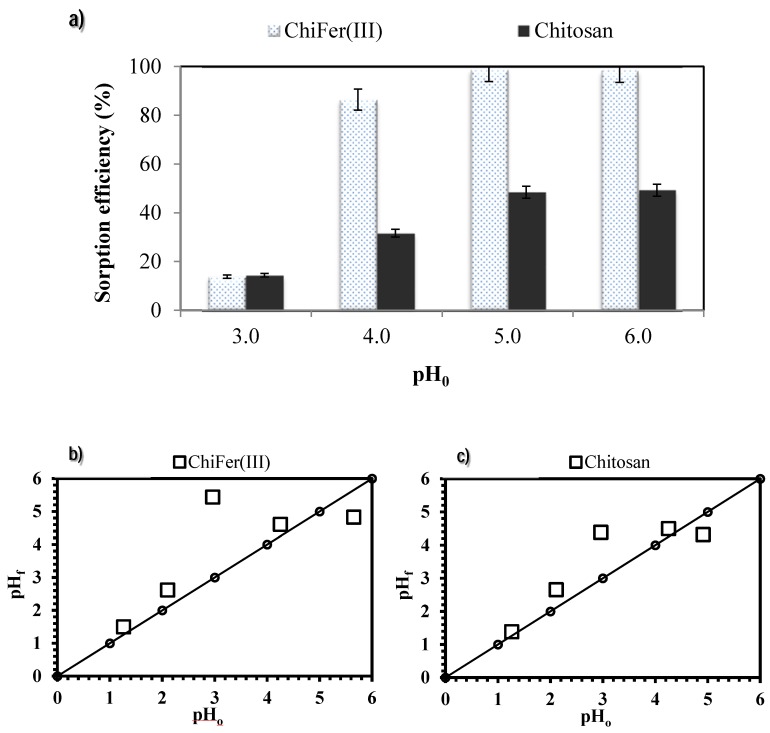
Influence of pH on neodymium removal. (**a**) Sorption efficiency. (**b**) Variation in pH using ChiFer(III) as sorbent. (**c**) Variation in pH using chitosan as sorbent. (T: 20 °C; sorbent dosage, SD: 1 g·L^−1^; agitation speed: 150 rpm; contact time: 72 h; C_0_: 9.4 mg·L^−1^).

Sorption performances of ChiFer(III) were compared with raw chitosan particles in the range of pH 1 to 6 (Figure 4a); at very acidic conditions the sorption is not favored since at pH below pH 3 some traces of iron are released into the solution, from pH < 2 the sorbents are completely dissolved (Figure S4), due to the natural hydrolysis of chitosan in acidic medium [49]. The best results and stability were obtained in the range of pH 4–6; the material is sufficiently stable and the introduction of iron into the chitosan matrix may enhance the sorption capacity of raw chitosan: at pH 5 the ChiFer(III) material increased by two times the removal achieved by chitosan particles (i.e., 98.8% and 49.3% respectively).

Figure 4b,c show the pH variation after metal sorption: when the initial pH was in the range 3–4, the equilibrium pH increased up to 4.4–5.0; for initial values of pH 5.0–6.5 the equilibrium pH tends to stabilize around pH 4.8–5.5. This type of “buffering effect” can be attributed to the acid– basic properties of chitosan; according to Sorlier et al. [50], the pKa of chitosan depends on parameters such as the deacetylation degree and the ionization extent of the polymer. The pKa for standard samples varies between 6.3 and 6.8; when the solution pH is below the pKa, chitosan tends to bind protons increasing the pH. Hence, at low pH values, the coordinating atoms in the sorbent (amino groups) are protonated which in contact with Nd species lead to repulsive electrostatic forces, limiting the sorption onto the sorbents materials.

### 3.3. Equilibrium Studies

Isotherms are critical in optimizing sorption processes since they help to describe the distribution of the sorbate between the liquid and solid phases at equilibrium. The correlation of data by theoretical or empirical equations is essential for the practical design of the sorption systems; different models have been proposed in the literature including Langmuir, Freundlich, and Sips equations, nevertheless, the fit of the experimental data does not mean that the principles of the models are verified, although it leads to a better interpretation of the sorption mechanism.

**Figure 5:**
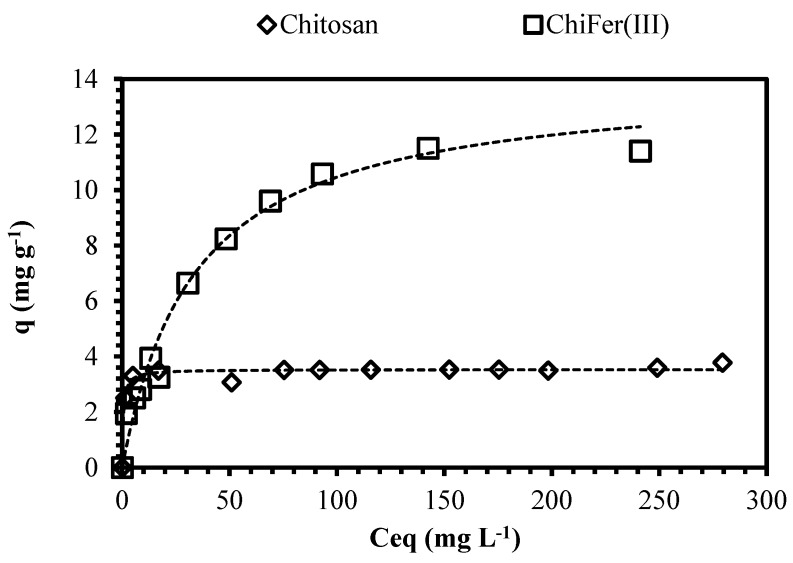
Isotherm plots for neodymium removal. (Solid line: Langmuir model; T: 20 °C; sorbent dosage, SD: 1 g·L^−1^; agitation speed: 150 rpm; contact time: 72 h; pH: 4; C_0_: 5–400 mg·L^−1^).

Figure 5 shows the impact of the metal concentration on the sorption uptake; increasing the neodymium concentration increases the metal removal progressively until a saturation plateau is reached (beyond which no further sorption can take place) [51]. At dilute metal concentrations (i.e., 5 mg·L^−1^) the sorbents allow removing of more than 60% of Nd; it meets the characteristics of a typical favorable sorption profile (asymptotic shape of the isotherm is consistent with the Langmuir equation), making these materials interesting for the treatment of dilute effluents.

Table 1 shows the fitting values of the Langmuir, Freundlich, and Sips models; the experimental data were fitted with Langmuir equations with better accuracy. Sips equation is a common adaptation of the Langmuir–Freundlich models; values for 1/ns close to zero are generally associated with heterogeneous sorbents, while values closer to 1.0 correspond to sorbents with relatively homogeneous binding sites [36]. Sorption capacity of ChiFer(III) is almost four times higher than raw chitosan particles, it is consistent with the values obtained in the pH study: the introduction of iron into the composite increases the removal of Nd (the maximum sorption capacities obtained are 3.5 mg·g^−1^ and 13.8 mg·g^−1^ for chitosan and ChiFer(III) respectively); taking into account that iron content in the composite is 0.33 g per gram of dried ChiFer(III), the contribution of iron is calculated as 30.9 mg Nd/g Fe, which is very close to the value reported by Tu et al. [4] using magnetic iron oxide (Fe_3_O_4_) particles as sorbent (Table 2).

Moreover, the configuration of the material under the form of spherical beads contributes to improving the handling of the sorbent for a fixed packed (column) system; it avoids the typical operational difficulties found for non-regular particles (as chitosan): (i) high head-loss and clogging effect; (ii) diffusional problems; (iii) reduction of the mass transfer performance due to a non-uniform arrangement of the bed in the column. The findings can be compared with those obtained by Wang et al. [24]; the authors evaluated calcium alginate under beads configuration and a novel hybrid gel (ALG-PGA: prepared from calcium alginate (ALG) and y-poly glutamic acid (PGA)); the results demonstrated that the introduction of PGA molecules into the alginate matrix, significantly enhanced the sorption capacity of the final sorbent. Thus, polymeric structures may improve the management of active materials and according to the functional groups may contribute to increasing metal uptake.

Table 2 shows a comparison of the maximum sorption capacities of several sorbents found in the literature; ChiFer(III) has a sorption uptake of the same order of magnitude. Although SiO_2_/CMCH material [42] has a higher performance, the simplicity of the manufacturing process and the hydro-dynamic ability for columns make ChiFer(III) beads a promising sorbent for neodymium removal from aqueous solutions.

### 3.4. Influence of Contact Time 

**Figure 6:**
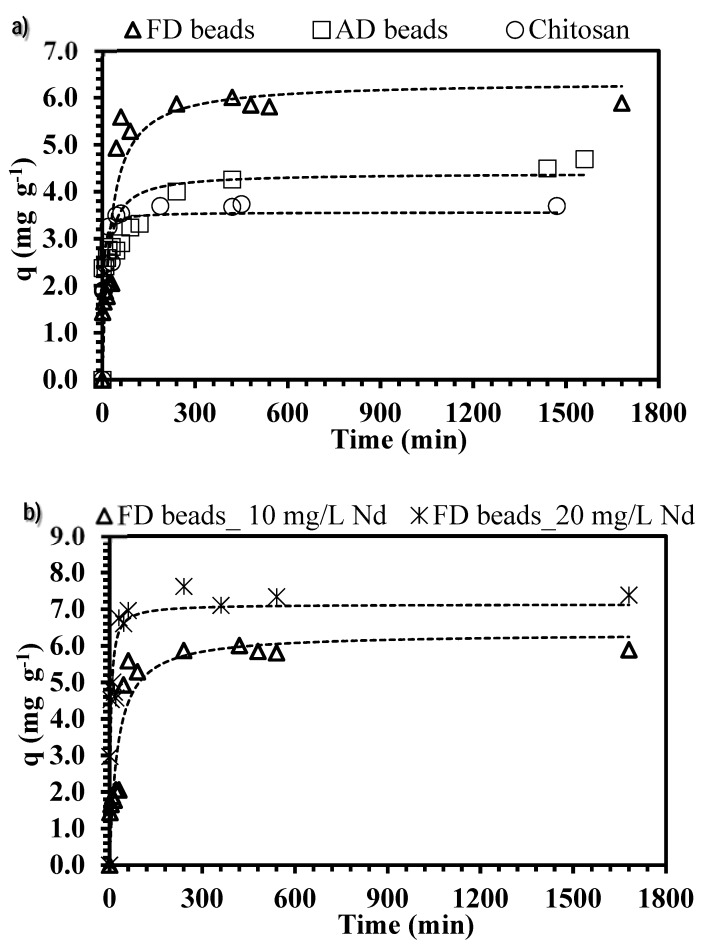
Effect of contact time on ChiFer(III) material performance. (**a**) Effect of drying method. (**b**) Effect of initial metal concentration. (Dashed line: Pseudo-second order rate equation (PSORE); T: 20 °C; sorbent dosage, SD: 0.5 g·L^−1^; agitation speed: 150 rpm; contact time: 72 h; pH: 4; C_0_: 10–20 mg·L^−1^).

Figure 6 shows the kinetic profiles for sorption of neodymium using freeze-dried beads (FD), air-dried beads (AD), and raw chitosan particles; in the literature, it was reported that the drying procedure may impact on the kinetic profiles [43]. Different techniques have been used for maintaining the original structures of hydrogels after drying. Borisova et al. [44] suggested that drying under supercritical CO_2_ conditions may improve the storage and lead to conserving the porosity of the manufactured materials (as a consequence, accessibility into active sites is not strongly modified); however it could be cost-expensive (in terms of energy) for drying large amounts of materials (at industrial scale production). In turn, freeze-drying and air-drying techniques (which are easy to operate at large-scale production) were compared in order to evaluate their contribution to the kinetic profiles.

ChiFer(III) beads and chitosan particles have a similar trend, and regardless of the type of drying, three pseudo steps can be considered: (i) an initial fast step that takes around 20 min for chitosan particles and 30–45 min for FD and AD beads (it represents 12%, 14%, and 31% of the total metal sorption using AD, chitosan and FD beads respectively); (ii) a second step which takes 3–6 h and accounts for an increase of 2%–13%; (iii) a third step which accounts for a lesser metal uptake (<5%). The difference in metal sorption (of each step) can be explained by the fact that the concentration gradient decreases progressively with the time: at longer contact time, the mass transfer is slower, since the driving force decreases.

It is noteworthy that kinetic uptakes are controlled by several mechanisms, including: (i) bulk diffusion; (ii) external diffusion (also called film diffusion); (iii) intraparticle diffusion; and (iv) reaction rate. Benettayeb et al. [54] pointed out that by maintaining a sufficient agitation speed and avoiding the settling of the sorbent, the contribution to the bulk diffusion could be neglected: this consideration was especially taken into account for performing the kinetic experiments and the system was kept continuously agitated (150 rpm).

As expected (Figure 6a), the time for achieving the equilibrium is shortened for chitosan due to the small particles size (0.5 mm < Sp < 1 mm) which results in more available surface area, impacting on the resistance to the external film diffusion and a greater number of active sites are quickly available. Moreover, the time required for FD is less than for AD beads (5 h for FD and 7 h for AD); it can be explained by the open structure of the sorbent, produced as a result of the freeze-drying technique, which improves the diffusion and the accessibility of neodymium into the active sites. Table 3 reports the comparison of the experimental sorption capacities at equilibrium with calculated values for both the pseudo-first order and the pseudo-second order rate equation models (PFORE and PSORE); the correlation coefficients confirm a better fitting with PSORE.

Additionally, the simplified equation of Weber and Morris, [37] (W&M) was tested for evaluating the contribution of the resistance in the intraparticle diffusion. The plots on Supplementary Materials Section (Figure S5) have three slopes (corresponding to the external surface diffusion, intraparticle diffusion, and reaction rate steps), which do not pass through the origin; this is an indication that intraparticle diffusion is not the unique mechanism that controls neodymium removal. Thus, K_p_ values were obtained from the slope of the linear portions of the second phase of the kinetics profiles, these do not show a significant trend (Table 3), but it is evident that the K_p_ value for FD beads is higher than for AD beads at the same operating conditions (this confirms the easy accessibility and a better diffusion is performed into the pores of FD material). Nevertheless, for chitosan particles, the initial slope (attributed to the external diffusion) is considerably steeper than the second step (attributed to the intraparticle diffusion); this verifies that intraparticle diffusion is not only the controlling step of the process.

Furthermore, increasing the initial concentration of neodymium (Figure 6b), increased the uptake in the initial and the second sections of the kinetic profiles: the contact time for achieving the equilibrium is reduced (4.5 h are required for reaching complete saturation plateau). It means that an increase of the metal concentration involves an increase of the concentration gradient between the solution and the internal/external surface of the ChiFer(III) material; as a consequence, the driving force increases which also involves a faster migration of ions and a higher sorption rate [45].

Moreover, Rorrer et al. [55] pointed out that a pore blockage mechanism may occur at low metal concentrations; the sorbed species flux is low, resulting in the accumulation of the sorbate species at the entrance to the porous network, which could finally block the external cavity of the pore. This mechanism was not completely evidenced in this study.

### 3.5. Dynamic System

In sorption processes, an important task for determining the feasibility of a promising sorbent is the capability of being used in a continuous system. Columns experiments are very useful to approach the real applications in the treatment of industrial effluents; although most of the results, currently present in the literature are reported on the batch system; the relevance of columns must be highlighted since the assessment of the monitoring parameters (such as flow rate, inlet metal concentration, pH, internal diameter of the column, and bed-depth of the packed sorbent) leads to improve the design and enhances the scaling-up of the sorption technique as a function of the sewage characteristics.

The simplicity of using a fixed amount of packed sorbent for treating large volumes of polluted waters (instead of a batch configuration) involves a better engineering conception of the material; the operating time could be easier optimized and the unnecessary treatment steps could be avoided (such as filtration of the loaded sorbent). According to the experimental data, the breakthrough curves were plotted for neodymium removal from dilute aqueous solutions, the sorption capacities were calculated until the breaking (q_BP_) and exhaustion (q_exp_) points.

**Figure 7:**
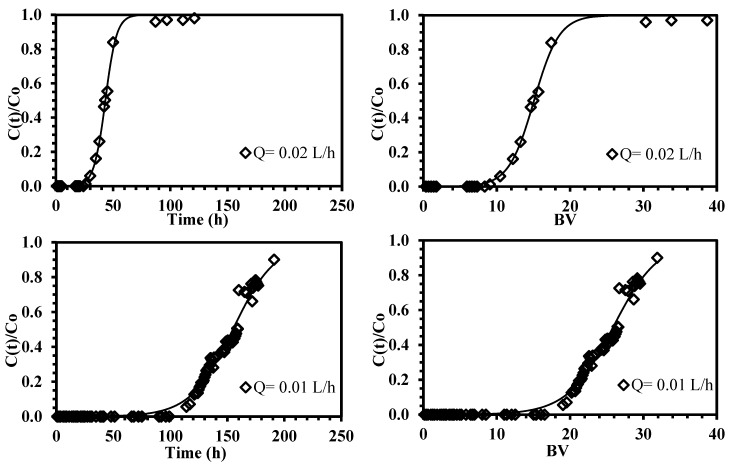
Continuous sorption of neodymium using ChiFer(III) material as fixed-packed sorbent. (**Left side**: Breakthrough curves as a function of time; **right side**: Breakthrough curves as a function of bed-volume; solid line: Thomas model; T: 20 °C; internal diameter of the column, Ø: 1.8 cm; bed depth: 23 cm; flow rate: 0.01–0.02 L·h^−1^; pH: 4; C_0_: 10.2 mg·Nd(III)·L^−1^).

Figure 7 shows the typical S-shape of the curves, by using two different flow rates (0.01 and 0.02 L·h^−1^); two common steps can be noted: (i) a first step where the neodymium species are totally sorbed; and (ii) a second step characterized by the progressive appearance of the metal, due to the continuous competition for occupying the active sites into the sorbent [56].

The breakthrough curves are similar in shape, but in terms of breakthrough time are substantially different; a decrease in the superficial velocity involves an increase of the effectiveness of the process; approximately 5% of the feed concentration is leached from the column after nine bed-volumes using a flow rate of 0.02 L·h^−1^; this result is improved when the flow rate is halved (i.e., 0.01 L·h^−1^), the sorption efficiency is increased from 25% to 79% and 19 bed-volumes are required for achieving the breaking point (Table 4). These differences are directly related to the residence time for mass transfer; when the higher superficial velocity is used, the external film diffusion is reduced, thus faster breaking and saturation times are produced. These findings are in agreement with the results obtained by Muhamad et al. [57] and Demey et al. [45] on heavy metal removal on using the biosorption process.

Additionally, the sorption capacity obtained in the exhaustion point (q_exp_) is two times lower of that obtained in the batch system (i.e., 13.76 mg·g^−1^ in batch system and 6.40 mg·g^−1^ in column system); this fact was attributed by Kleinübing et al. [58] to the resistance to the film diffusion which was particularly active in dilute concentrations (below 0.5 mmol·L^−1^), on the continuous removal of copper and nickel from aqueous solutions with marine alga species; the mass transfer could be improved by reducing the flow rate.

ChiFer(III) material was evaluated in this work through three consecutive sorption/desorption cycles (in the batch system using 0.1 mmol·L^−1^ neodymium solutions, Figure S6); the performance of the beads drastically decreased from the third sorption cycle (i.e., it drops from 99.1% in the first cycle to 30.3% in the third cycle). It is noteworthy that ChiFer(III) was previously tested for boron recovery from seawater in several sorption–desorption cycles; the stability of the material and its performance was not affected under extreme saline conditions [23]. Thus, further studies will be performed in order to enhance the reusability of the sorbent with real effluents (containing boron and neodymium), which will be the scope of a future work. In order to verify the selectivity sequence of the sorbed metals, an equimolar solution of boron and neodymium (0.1 mmol·L^−1^) was passed into the column; this influent was prepared to approach a real application of metal recovery from dilute waste waters of permanent magnets (Nd-Fe-B).

**Figure 8:**
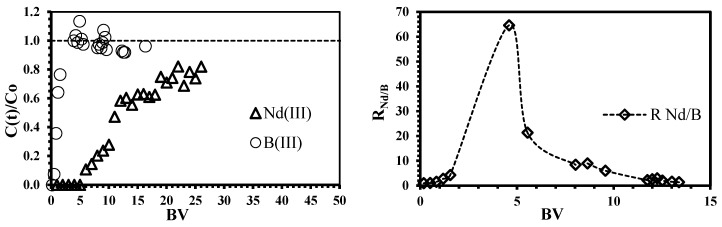
Simultaneous sorption of neodymium and boron using ChiFer(III) material as fixed-packed sorbent. (**Left side**: Breakthrough curves as a function of Bed-volume (BV), dashed line: overshooting guide line; **right side**: Separation coefficient R_Nd/B_ as a function of BV; dashed line: experimental trend; T: 20 °C; internal diameter of the column, Ø: 1.8 cm; bed depth: 23 cm; flow rate: 0.01 L·h^−1^; pH: 4; C_0_: 0.1 mmol·L^−1^).

The breakthrough curves from the binary mixture are plotted in Figure 8; three relevant features are highlighted: (i) the first step of the sorption curve is more prolonged for neodymium than for boron, which is evidence of the higher selectivity toward Nd ions; (ii) a significant reduction of the bed-volumes on neodymium appearance was obtained (in comparison with single metal solution, the bed-volume values were reduced from 19 to 3.9); the process becomes less efficient, this is a consequence of the strong competition of species for active sites into the sorbent material; iii) boron in presence of neodymium is eluted from the column in a shorter time (5% of the feed boron concentration is leached after 0.5 bed-volumes), thus, boron overshoot is produced: the outlet concentration is higher than inlet concentration (i.e., C/C_o_ > 1). The overshoot in a column system is based on the proper nature of the sorption itself, the sorbent has a limited sorption capacity and the species with lower affinity are pushed off and displaced by the species of higher affinity [59]; this phenomenon is in agreement with Sag and Kutsal, [60].

Although the sorption of boron species (as co-ions) is almost negligible, due to the higher selectivity to Nd ions, the removal uptake of neodymium is inhibited (the sorption performance decreases from 79% in single solution to 49% in binary system, Table 4 and Table S1). Some authors have suggested that the non-binding co-ions contribute to balancing the negative charges on the biomass surface (i.e., the electro-neutrality principle); this could inhibit electrostatically the binding of the main metal even though the co-ion is not bound to the sorbent [61]. The sorption mechanism onto iron surface was reported by Smith and Ghiassi, [62] and Grossl et al. [63] by using iron particles and goethite, respectively, as sorbents for chromate removal; the modeling results suggested the formation of monodentate inner-sphere complexes onto iron particles and bidentate inner-sphere complexes onto goethite. However, the interaction between the iron(III) hydroxide and boron species occurs similarly to an esterification reaction: both boric acid and borate ions can react with the hydroxide compounds to form the boric and borate esters [64]; this mechanism can only take place if the distance between adjacent OH^−^ groups in the Fe(OH)_3_ structure are similar to those of OH^−^ groups of boron species [23,64].

**Figure 9:**
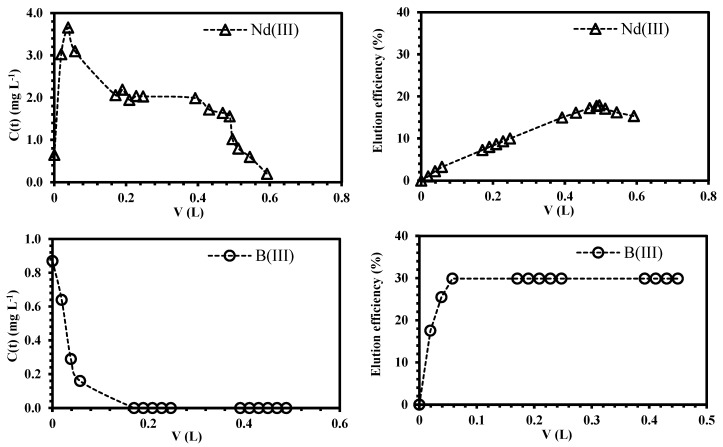
Simultaneous desorption of neodymium and boron from loaded ChiFer(III) material as fixed-packed sorbent. (**Left side**: Recovery metal concentration as a function of the volume (L), dashed line: desorption trend; **right side**: Elution efficiency as a function of the volume (L); dashed line: efficiency trend; T: 20 °C; internal diameter of the column, Ø: 1.8 cm; bed depth: 23 cm; flow rate: 0.01 L·h^−1^; Eluent: demineralized water at pH: 3.5).

The separation factor for equimolar solutions (R_Nd/B_) was plotted as a function of bed-volume (Figure 8). The values of R_Nd/B_ >>1 confirm the higher neodymium selectivity of the sorbent; as expected, the point of maximum separation degree corresponds to 4.6 bed-volumes, which is also the point of the maximum overshooting of boron; consequently, the affinity of ChiFer(III) follows Nd >>B. The regeneration of the column was carried out with dilute HCl solution (demineralized water at pH 3.5); Figure 9 shows that only 0.6 L of eluent is enough for metal recovery: 20% of the total sorbed Nd and 30% of boron (it represents the remaining boron which was not previously overshot). The possibility of recovering the sorbed metals from the loaded ChiFer(III), makes this sorbent interesting for future separation of neodymium from real effluents.

## 4. Conclusions

ChiFer(III) material is a promising and efficient sorbent for neodymium recovery from effluent waters. The incorporation of iron(III) hydroxide into the chitosan matrix, improves the sorption efficiency of the resulting material. The maximum sorption capacities obtained were 3.5 mg·g^−1^ and 13.8 mg·g^−1^ by using chitosan and ChiFer(III), respectively. The Sips model and PSORE fitted accurately the sorption isotherms and the kinetic experimental data, respectively. Column experiments demonstrated the feasibility of recycling the sorbents; the regeneration of the packed-bed can be performed with only 0.6 L of eluent (demineralized water at pH 3.5). The evaluations with simulated effluent of boron and neodymium demonstrated the higher selectivity of the sorbent toward Nd ions, which is useful for future evaluations with real wastewater effluents; the faster overshooting of boron in the columns enhances the separation of neodymium. The continuous sorption data can be described by the Thomas equation.

## Figures and Tables

**Table 1:** Langmuir, Freundlich, and Sips constants of ChiFer(III) and chitosan particles.

Experimental		Langmuir			Freundlich			Sips			
Sorbent	q_exp_ (mg·g^−1^)	q_max_ (mg·g^−1^)	b (L·mg^−1^)	r^2^	K_F_ (mg^1−1/n^ g^−1^·L^1/n^)	n	r^2^	q_max_ (mg·g^−1^)	Ks (L·mg^−1^)	ns	r^2^
Chitosan	3.77	3.54	1.91	0.971	2.78	20.44	0.957	3.51	1.75	0.80	0.966
ChiFer(III)	11.51	13.76	0.03	0.973	1.65	2.63	0.922	13.80	0.03	1.00	0.969

**Table 2:** Comparison of sorption capacities for several sorbents.

Sorbent	pH Range	qmax (mg·g^−1^)	Authors
*C. colliculosa yeast*	1.5	10.0	Vlachou et al. [52]
*K. marxianus yeast*	1.5	12.0	Vlachou et al. [52]
*D. hansenii yeast*	1.5	10.0	Vlachou et al. [52]
ChiFer(III) beads	4.0–5.0	13.8	This work
Cysteine-functionalized chitosan magnetic particles	6.0	15.3–17.1	Galhoum et al. [25]
Magnetic iron oxide (Fe_3_O_4_)	8.2	24.8	Tu et al. [4]
Ion imprinted polymer	7.0–7.5	35.2	Guo et al. [53]
SiO_2_/Carboxymethyl chitosan (CMCH)	6.9	53.0	Wang et al. [42]

**Table 3:** Kinetic parameters of sorbent materials.

Experimental		Pseudo-First Order Rate Equation (PFORE)			Pseudo-Second Order Rate Equation (PSORE)			Weber and Morris Equation (W&M)
Sorbent	q_exp_ (mg·g^−1^)	K_1_ (min^−1^)	q_1_ (mg·g^−1^)	r_2_	K_2_ (g·mg^−1^·min^−1^)	q_2_ (mg·g^−1^)	r^2^	K_p_ (mg·g^−1^·min^−1/2^)
Air-dried, AD (C_0_ = 10 mg·g^−1^)	4.50	5.95 × 10^−2^	3.96	0.455	1.69 × 10^−2^	4.39	0.619	0.14
Freeze-dried, FD (C_0_ = 10 mg·g^−1^)	5.89	2.78 × 10^−2^	5.93	0.908	6.16 × 10^−3^	6.34	0.887	1.24
Chitosan (C_0_ =10 mg·g^−1^)	3.74	0.177	3.44	0.757	0.11	3.56	0.851	2.81 × 10^−2^
FD (C_0_ =20 mg·g^−1^)	7.39	0.129	6.88	0.778	3.9 × 10^−2^	7.13	0.885	0.64

**Table 4:** Sorption parameters for continuous system using ChiFer(III) material as sorbent.

Experimental						Thomas Parameters		
Sorbate	Q (L·h^−1^)	q_exp_ (mg·g^−1^)	Sorption Efficiency (%)	q_BP_ (mg·g^−1^)	Bed-Volume (BV_BP_)	q_T_ (mg·g^−1^)	K_T_ (L·h^−1^·mg^−1^)	r^2^
Nd	0.02	4.02	24.97	2.12	9.06	3.46	1.98 × 10^−2^	0.984
Nd	0.01	6.40	79.17	4.79	19.03	6.55	5.27 × 10^−3^	0.981

## Supplementary Materials

The supplementary materials are available online at http://www.mdpi.com/2073-4360/10/2/204/s1.
