# Supplementary material for: Neodymium Recovery by Chitosan/Iron(III) Hydroxide [ChiFer(III)] Sorbent Material: Batch and Column Systems
Source: Polymers (Basel). 2018 Feb 19;10(2):204. doi: 10.3390/polym10020204 (PMC6414884; doi:10.3390/polym10020204)
Supplement: Supplementary file 1 [file polymers-10-00204-s001.docx]

**Supplementary Materials: Neodymium Recovery by Chitosan/Iron(III) Hydroxide [ChiFer(III)] Sorbent Material: Batch and Column Systems**

Hary Demey ^1,2^*, Byron Lapo ^1,3^, Montserrat Ruiz ^4^, Agustin Fortuny ^4^, Muriel Marchand ^2^ and Ana M. Sastre ^1^


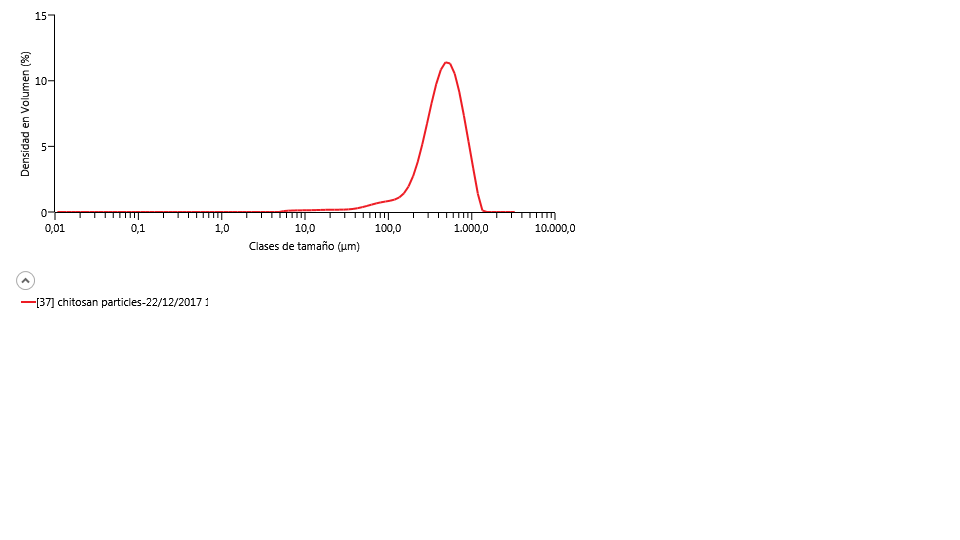


**Size (μm)**

**Density in Volume (%)**

**Figure S1.** Average particle size of chitosan.


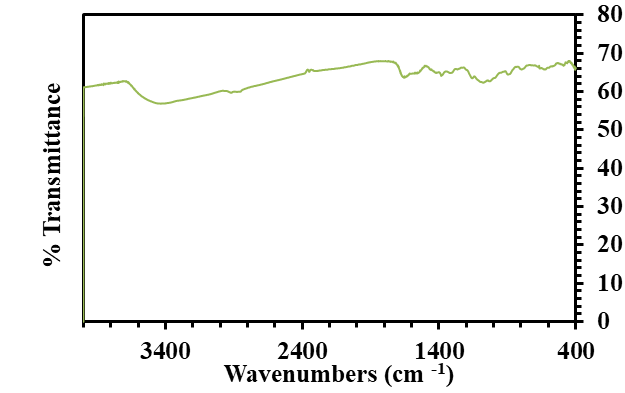


**Figure S2.** FTIR analyses of ChiFer(III) sorbent.

**Figure S3.** Metal species as a function of pH.


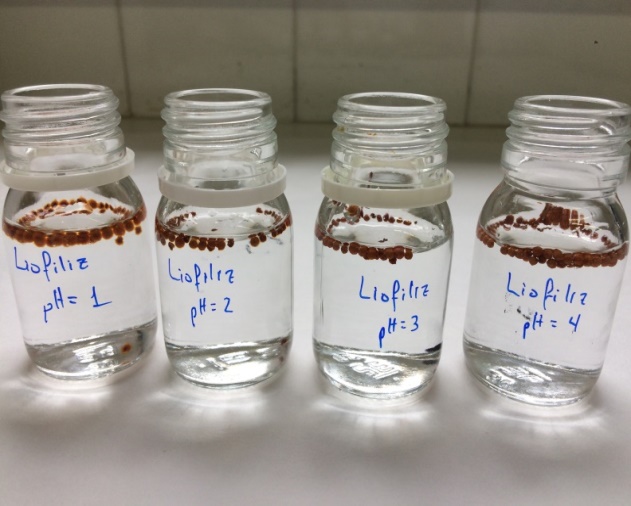

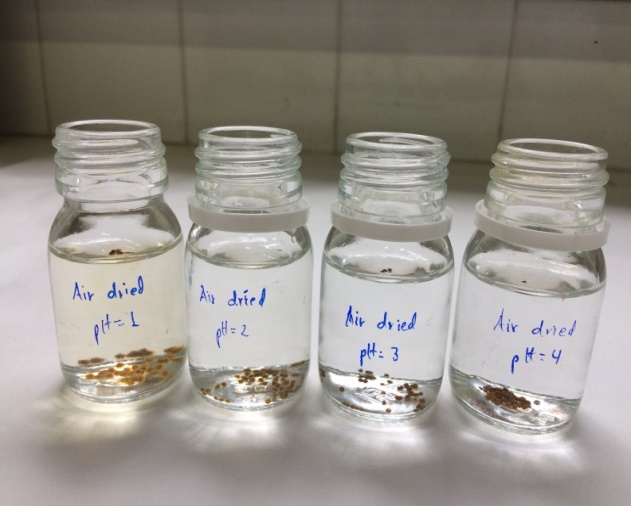

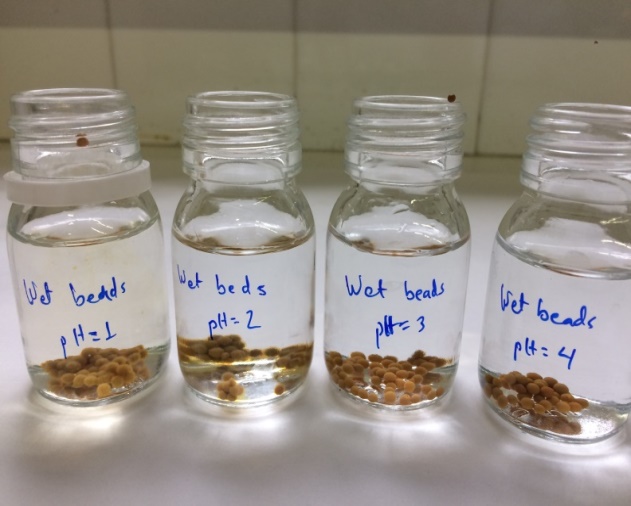

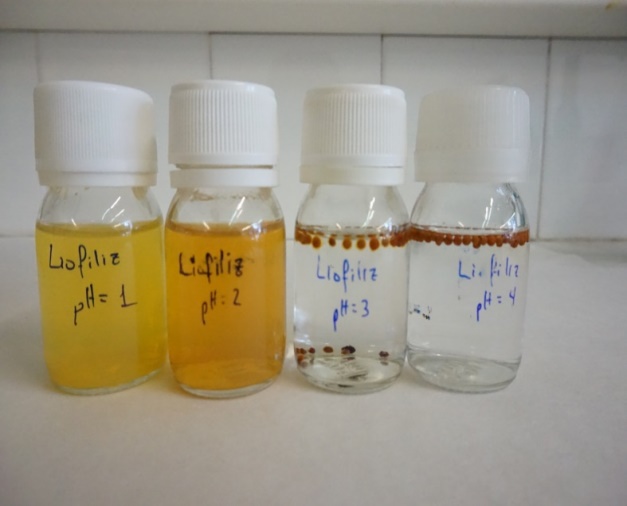

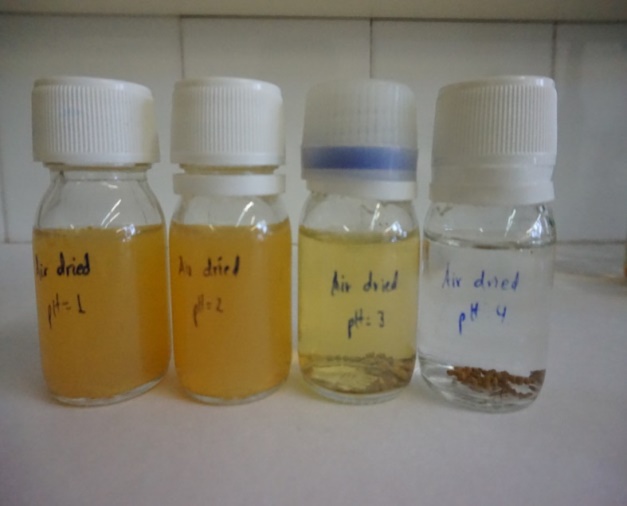

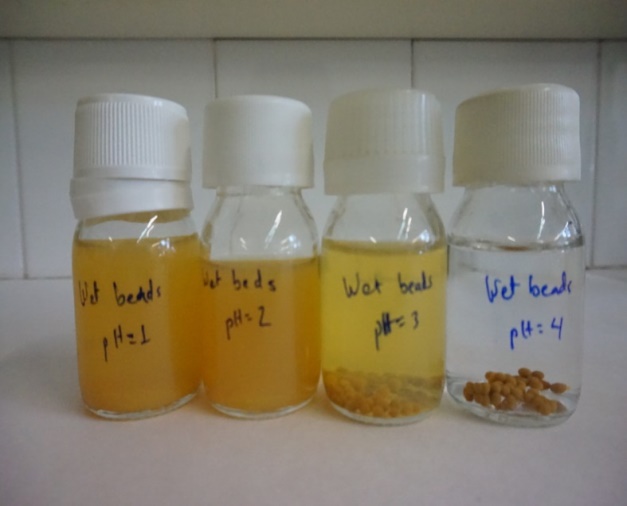


**a)**

**b)**

**c)**

**c.1)**

**b.1)**

**a.1)**

**Figure S4.** Stability of the ChiFer(III) beads as a function of pH. Left side: before sorption (Figure S4a wet beads. Figure S4b freeze dried beads. Figure S4c air dried beads). Right side: after sorption (Figure S4a1 wet beads. Figure S4b1 freeze dried beads. Figure S4c1 air dried beads).


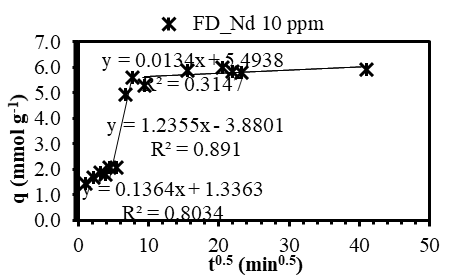

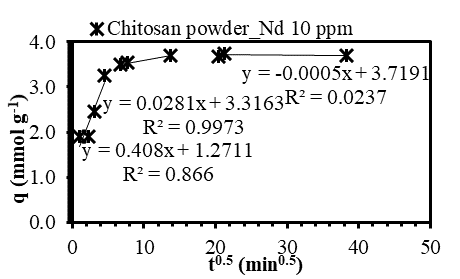

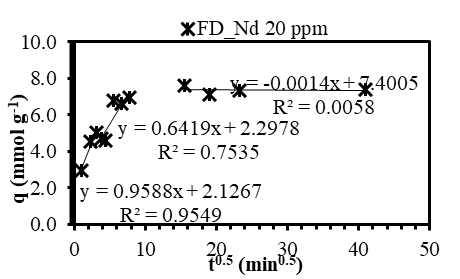

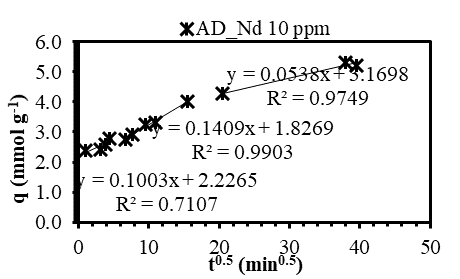


**Figure S5.** Intraparticle diffusion coefficients using freeze dried (FD), air-dried (AD) and chitosan particles as sorbents.

**Figure S6.** Sorption-desorption cycles using ChiFer(III) beads as sorbent.

**(**T: 20 °C; sorbent dosage, SD: 1 g L^-1^; agitation speed: 150 rpm; contact time: 72 h; pH: 4; C_0_: 0.1 mmol L^-1^).

**Table S1.** Sorption parameters for simultaneous removal of neodymium and boron from aqueous solutions.

| Sorbate  Nd/B | Q  (L h^−1^) | q_exp_  (mg∙g^−1^) | Sorption efficiency (%) | q_BP_  (mg∙g^−1^) | BV_BP_ |
| --- | --- | --- | --- | --- | --- |
| Nd | 0.01 | 2.45 | 49.0 | 1.12 | 3.91 |
| B |  | 0.03 | 9.44 | 0.01 | 0.50 |
